# Comparison of Vernier Acuity Measured with Three Different Water-induced Blur Simulation Methods

**DOI:** 10.18502/jovr.v20.13887

**Published:** 2025-07-21

**Authors:** Vivek Suganthan Ramasubramanian, Aiswaryah Radhakrishnan

**Affiliations:** Department of Optometry, Faculty of Medical and Health Sciences, SRM Medical College Hospital and Research Centre, SRM Institute of Science and Technology, Kattankulathur, Tamil Nadu, India

**Keywords:** Hyperacuity, Underwater Blur, Underwater Vision, Vernier Acuity, Vision

## Abstract

**Purpose:**

Human vision is subnormal in an aquatic environment, and studies have used different methods to measure visual functions with water-induced blur (WIB). In this study, we compared vernier acuity measured using three different WIB simulation methods.

**Methods:**

Sixty young adults (20 in each group) with best-corrected visual acuity of ≥ 6/6 participated in the study. Three different methods, one for each study group, were used to simulate WIB in order to measure the vernier acuity. The methods comprised M1: a glass tank filled with water containing a wave motor to produce waves, M2: a sprinkler with uncontrolled water splash against the glass, and M3: a sprinkler with controlled water splash against the glass. For each of the three methods, vernier acuity was measured binocularly (three trials of 50 presentations each) both at baseline (without simulated WIB) in the absence of WIB and under simulated WIB. This was conducted using FrACT presented on the Display++ monitor at a distance of 2 meters from the participant. The vernier target consisted of two vertical lines (1 x 15 arcmin) with a vertical separation of 0.5 arcmin.

**Results:**

The mean baseline vernier acuity (arcsec) was found to be similar (F^[2, 57]^ = 0.20, *P *= 0.82) among all three groups (M1: 13.28 ± 5.84, M2: 14.44 ± 6.34, M3: 14.05 ± 3.28). Vernier acuity with simulated WIB was least degraded with M1 (19.84 ± 8.40) and more degraded with M2 (288.74 ± 56.61), followed by M3 (49.14 ± 20.13). One-way ANOVA revealed a significant difference among the three methods (F [2, 57] = 354.72, *P* < 0.001).

**Conclusion:**

Our results suggest that the impact of simulated WIB on vernier acuity is not comparable due to differences in the strength of blur and the varied spatial and temporal properties of different simulated WIB methods. This emphasizes the need to develop a blur metric specific to WIB to objectively quantify its effect on different visual functions.

##  INTRODUCTION

The human visual apparatus is a highly robust system that compensates for aspects such as changes in illumination and color,^[1]^ intrinsic and extrinsic blur,^[2,3,4]^ as well as motion and motion-induced blur.^[5]^ It also possesses neural mechanisms for the rapid and efficient perception and recognition of objects and faces, often without requiring conscious effort.^[6]^ In addition, exposure to even short durations of newer and complex blur can result in an adaptation effect, which is observed as an improvement in visual functions such as acuity,^[7]^ contrast sensitivity,^[7,8]^ and even blur thresholds.^[9,10,11,12,13]^ These compensating mechanisms provide favorable outcomes in eyes with optical abnormalities, such as keratoconus or cataract, where improved visual performance is observed in diseased eyes compared to normal eyes exposed to the same amount of blur.^[4,11]^

Blur underwater is not uncommon and is encountered by swimmers and divers. When the human eye comes into contact with the water interface, the corneal power is negated.^[14]^ Unlike aquatic or amphibious animals, human eyes do not possess special adaptive mechanisms, such as altered corneal structure or crystalline lens shape, that enable clear vision in both air and water.^[15]^ Adaptation to such a severely defocused image is unlikely in terrestrial beings such as humans due to neural adaptation mechanisms alone.^[4,16]^ Underwater blur is the blur that is produced when the human eye directly interacts with the underwater environment.

A study revealed that Moken children possess superior underwater vision, which is twice as good as that of normal European children, thanks to pupil constriction and accommodation.^[8]^ Another study demonstrated that visual training enhanced underwater visual acuity in normal European children, attributed to pupil constriction and accommodation.^[7]^ When the human eye is in an underwater environment, even with the use of protective eyewear that restores the cornea's refractive power, vision is still affected by different factors. These factors include the turbidity of water,^[17]^ reduced illumination,^[8]^ and non-symmetric distortions introduced by random wave patterns, ripples, and bubbles with air pockets that have a different refractive index.^[18]^

**Figure 1:**
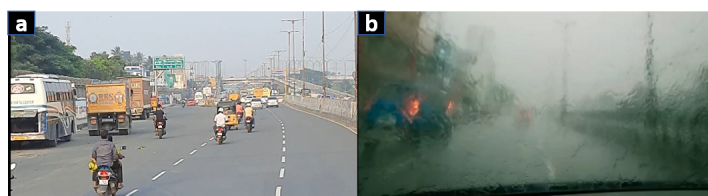
A view through the windshield of a car on (a) a normal day and (b) a rainy day.

Besides swimmers and divers, water-induced blur (WIB) is also commonly observed while driving on a rainy day [Figure 1], with water splattering on the windshield/screen. This can produce sharp intensity changes that can severely impair visual performance. In this study, simulated water-induced blur (SWIB) is defined as the blur experienced when a person sees any object or stimulus through a water medium. A study has shown that visual functions such as visual acuity, grating acuity, and contrast sensitivity deteriorate severely in the presence of SWIB.^[19]^

The presence of underwater blur adversely affects visual functions such as pupil size and eye movements.^[20]^ Studies have shown that underwater visual acuity^[8,21]^ and contrast sensitivity^[8,19]^ decrease by approximately 1.1 logMAR and 2 log units, respectively, due to underwater blur. The values reported across the studies vary due to differences in the methods used to simulate WIB. Hence, it is crucial to assess the effect of WIB as accomplished using different techniques.

Vernier acuity is a hyperacuity function that involves detecting the positional offset between stimuli and has implications for depth perception and related functional activities.^[22]^ The site of hyperacuity processing is presumed to be cortical, because the sensitivity to local position information exceeds the optical and photoreceptor sampling limits imposed by the eye.^[23]^ Research shows that vernier acuity is affected by stimulus parameters such as contrast and size,^[24]^ patient's age, photoreceptor density,^[25]^ and training.^[26,27]^ Hyperacuity largely depends on cortical processing and is highly resistant to image degradation, making it an important test of visual function in patients exhibiting optically degraded or partially opaque media.^[28]^ Additionally, the random wave pattern that occurs in the underwater environment can cause lateral displacement of the target, which may also affect vernier acuity. Therefore, it is crucial to understand the impact of WIB on the vernier acuity threshold in humans.

Understanding changes in visual performance with different SWIB methods helps evaluate how visual functions differ with each of these methods, and standardizing the methodology will aid in developing techniques to improve underwater visual performance. Hence, in this study, we investigated changes in vernier acuity among young adult humans when subjected to three different WIB simulation methods.

##  METHODS

### Participants

Sixty healthy adults (20 in each group), with a mean age of 21.2 ± 0.8 years, participated in the study. All participants were included after a comprehensive ocular examination and possessed normal or corrected-to-normal vision (6/6 or better visual acuity). None of the participants had > 1.00 D of cylinder in either eye and displayed no ocular or systemic abnormalities. The participants consisted of either students or staff from the SRM Medical College Hospital and Research Centre. The study was conducted in accordance with the Declaration of Helsinki, and all participants provided informed consent under the protocol approved by the Institutional Ethics Committee, SRM Medical College Hospital and Research Centre, SRMIST, India (Ethics Clearance Number: 1778/IEC/2019). Before being tested, the participants were familiarized with the vernier acuity experimental procedure, and sufficient demonstrations were provided to ensure they understood the experiment.

### Apparatus

**Figure 2:**
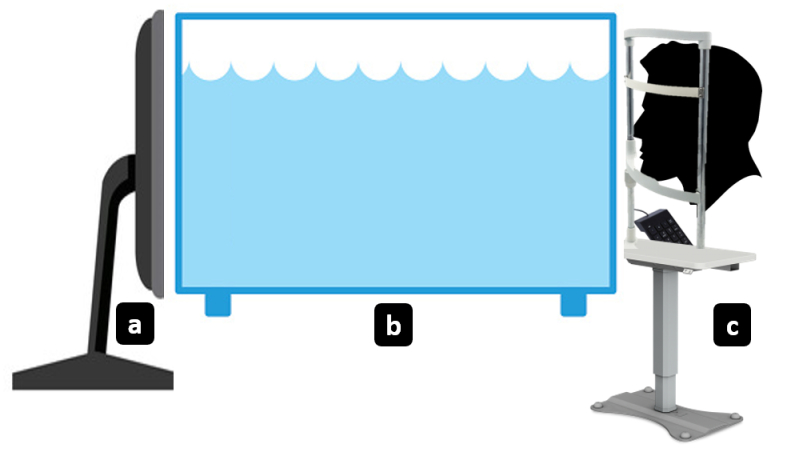
The experimental setup showing (a) stimulus display system, (b) water-induced blur simulation system, and (c) measurement system.

The experimental setup consisted of three units: a stimulus display system, a WIB simulation system, and a measurement system [Figure 2]. The stimulus display system consisted of a calibrated Display++ monitor (Cambridge Research Systems, UK) with a resolution of 1920 × 1080, which was controlled by a Windows laptop. The measurement system included a numeric keyboard synchronized for response acquisition. The stimulus was viewed binocularly with a natural pupil at a viewing distance of approximately 200 cm in dark illumination. Participants were seated in an ergonomic, height-adjustable chair with head and chin support, facing the monitor frontally.

Three different WIB simulation systems were developed and compared in this study.

**Figure 3:**
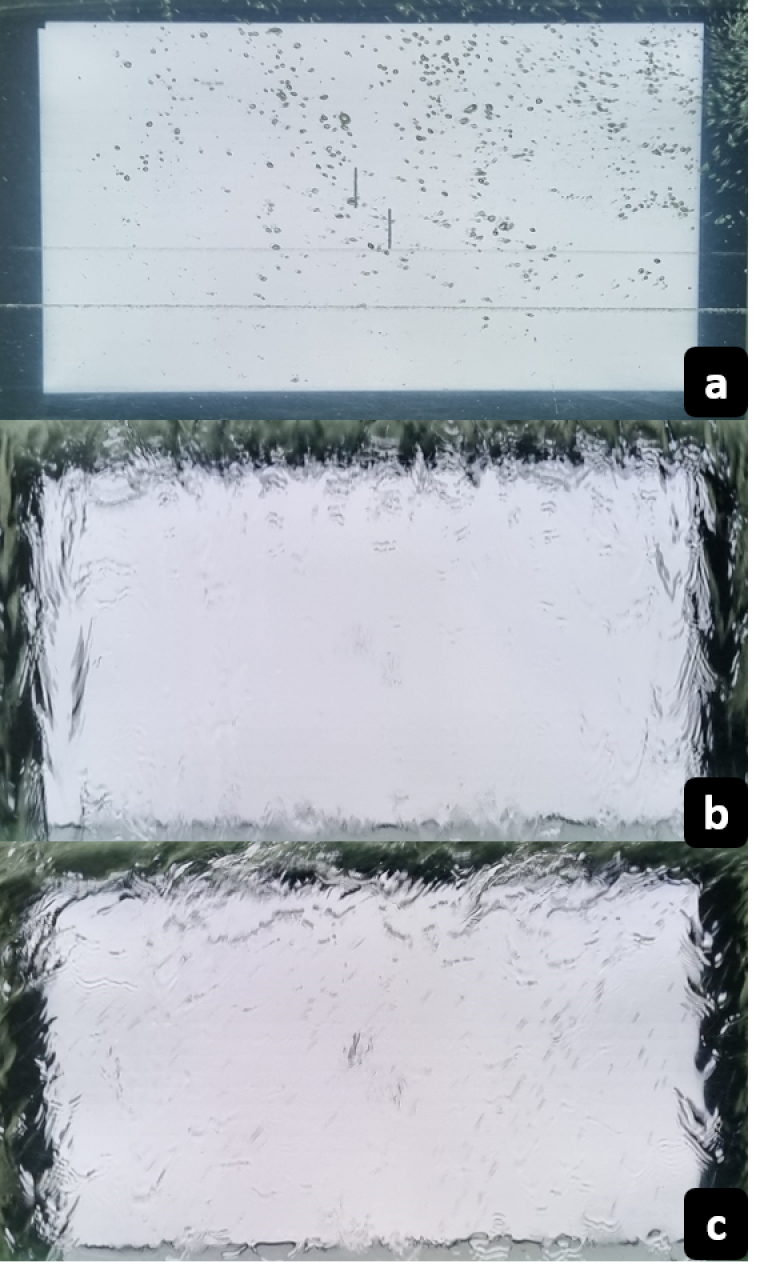
Experimental setup of three different water-induced blur simulation methods: (a) M1: Glass tank filled with water containing wave motor to produce waves, (b) M2: Sprinkler with uncontrolled water splash against the glass, and (c) M3: Sprinkler with controlled water splash against the glass.

**Figure 4:**
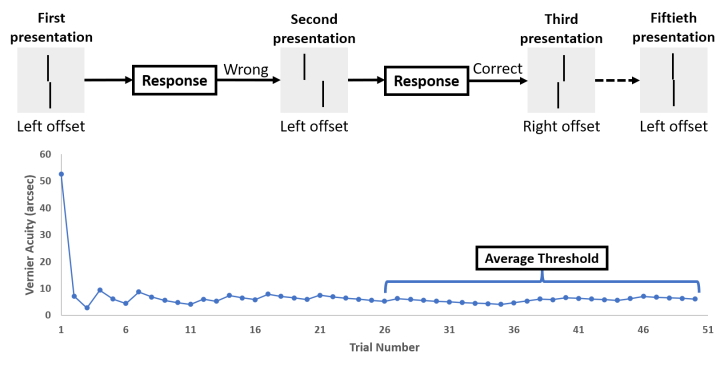
Estimation of vernier acuity using the best PEST procedure in FrACT. The participant indicated the upper bar's horizontal offset direction relative to the lower bar using a numeric keypad (4-left & 6-right). The graph shows a sample vernier acuity trial consisting of 50 stimulus presentations.

#### Method 1 (M1)

The simulation system consisted of a transparent glass tank measuring 100 x 80 x 50 cm (L x W x D), with a thickness of 12 mm, filled with water to a height of 46 cm. The tank was made up of crown glass with a refractive index of 1.52 and a transparency of 96.4%. There was no significant difference in transmission properties with the presence or absence of water in the tank. A commercial wave maker (25W, 20000 L/H) and a commercial air pump (2.5W, 180 L/H), strategically placed in the inner periphery of the glass tank, were used to generate random wave patterns (as usually observed on the water surface) and bubbles. On either side of the water tank, the stimulus display system and the measurement system were positioned. The entire stimulus display system was visible through the water tank. As shown in Figure 3a, the placement of the wavemaker and air pump did not conceal the stimuli.

#### Method 2 (M2)

To simulate WIB, a commercial sprinkler was used to splash water against a transparent glass tank placed between the participant and the monitor [Figure 3b]. In this case, the blur produced is uncontrolled, as the flow of water against the tank is not evenly distributed. This blur can be compared to the one experienced while driving a car on a rainy day.

#### Method 3 (M3)

The WIB simulated in this method [Figure 3c] was similar to Method 2. Evenly spaced holes with a diameter of 2 mm were drilled in a PVC pipe, and water was splashed against a transparent glass tank. The blur produced is similar to Method 2, except that it is now controlled since the water flow is evenly distributed.

### Stimuli

The Freiburg Visual Acuity and Contrast Test (FrACT) version 3.10.5^[29]^ was used to present the vernier acuity stimulus. The vernier target consisted of two vertical lines, each subtending 1 × 15 arcmin, with a vertical separation of 0.5 arcmin at 50% optotype contrast.

### Procedure

The vernier acuity stimulus was presented on the Display++ monitor placed in front of the participant. The participant was asked to detect the horizontal separation and indicate their binary decision using the numeric keypad. FrACT employs the best PEST (parameter estimation by sequential testing) procedure to determine vernier acuity, as shown in Figure 4. PEST is an adaptive psychophysical procedure that estimates sensory thresholds and other psychometric functions. The stimulus intensity after each trial is based on the participant's response, which reduces the number of trials required to converge on the true threshold. Vernier threshold acuity is expressed using the minimum angle of resolution (in arcsec), with lower values indicating better acuity. Each stimulus was displayed for a maximum of 30 seconds. Each participant made three sets of judgments on 50 trials each, and the average of the three sets was considered their vernier threshold acuity. Measurements were taken binocularly. Specifically, 20 participants were recruited to participate in the experiment using each method. Baseline vernier acuity (without SWIB, indicating the absence of WIB) was measured for all three methods with the display positioned 2 meters from the participants. On the other hand, vernier acuity with SWIB was measured at a distance of 2 meters from the display by placing a transparent glass tank between the participant and the display. The order of testing the conditions (with and without SWIB) was randomized for each participant. Participants were given sufficient breaks as needed.

### SWIB Quantification

**Figure 5:**
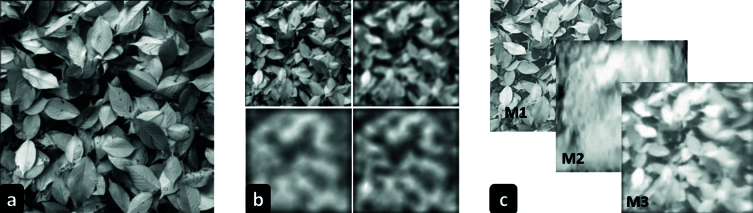
(a) Greyscale test image, (b) images with spherical blur (from top left: 1 D, 3 D, 7 D, and 4 D), and (c) images with three different water-induced blur simulation methods (M1: A glass tank filled with water, M2: A sprinkler with uncontrolled water splash, M3: A sprinkler with controlled water splash).

The WIB simulated in our study was quantified using the Structural Similarity Index Metric (SSIM), an image quality metric that evaluates the structural similarity of two images by considering luminance, contrast, and structural information.^[30]^ A greyscale test image [Figure 5a] of 500 × 500 px with 1/f properties was selected according to the natural statistics of images. The spherical blur (SB) [Figure 5b] was simulated in MATLAB by convolving the test image with the point spread function of the corresponding defocus. A total of 20 images were simulated with defocus values ranging from 0 D to 5.00 D in 0.25 D steps and then used for further analysis.

The previous setup mentioned in Figure 3 was used to capture images with SWIB. The test image was displayed on a Display++ LCD monitor, positioned at one end of the water tank. A video was recorded for 1 minute using a DSLR camera, and 20 random images [Figure 5c] were subsequently extracted from the recorded video for further analysis.

The SSIM was calculated using established MATLAB functions by comparing images with SB to those with SWIB. The resulting SSIM scores ranged from 0 to 1, with higher scores indicating greater similarity between the two images, suggesting that they are dioptrically equivalent.

### Analysis

The data were exported from the FrACT application to a Microsoft Excel spreadsheet. The normality of the data was evaluated using the Shapiro-Wilk test, which indicated that the data were normally distributed. Therefore, parametric tests were used for the remaining analyses. To describe the parameters, we used the mean and standard deviation. The comparison of mean vernier acuity between baseline and SWIB conditions was conducted using a paired *t*-test, and the mean vernier acuity among the three simulation methods was analyzed using one-way ANOVA. All statistical analyses were performed using Microsoft Excel and IBM SPSS Statistics for Windows, Version 20.0 (IBM Corp, Armonk, NY, US). *P*-values < 0.05 were considered statistically significant.

##  RESULTS

### Vernier Acuity Based on Three Different Methods

**Figure 6:**
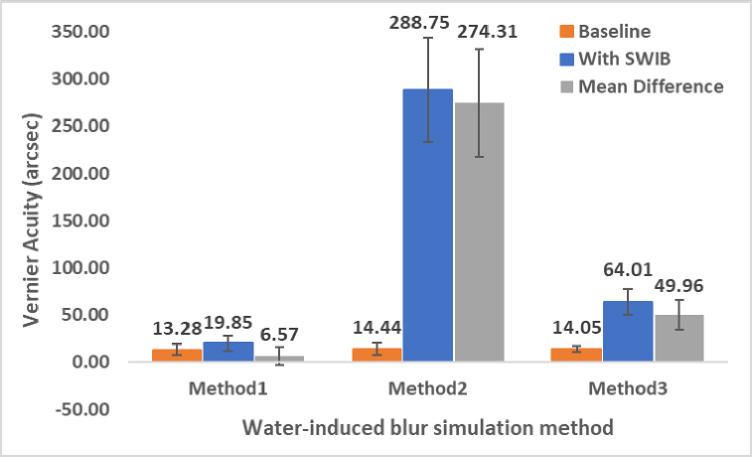
Vernier acuity (arcsec) compared among three different water-induced blur simulation methods. Error bars indicate standard deviation.

The changes in vernier acuity threshold (in arcsec) at baseline and with SWIB across three methods are shown in Figure 6. Lower values indicate better vernier acuity, while higher values indicate worse vernier acuity. The mean vernier acuity threshold at baseline across the three methods was found to be similar, as shown in Table 1. There was no significant difference in baseline vernier acuity among the three methods (F [2, 57] = 0.20, *P *= 0.82). Regardless of the method used, vernier acuity deteriorated significantly in the presence of SWIB [Table 1] compared to baseline. However, this deterioration was most evident between baseline and SWIB with M2, followed by M3 and M1. One-way ANOVA revealed a significant difference among all three groups (F [2, 57] = 354.72, *P* = 0.00).

Each of the three groups consisted of entirely different participants, with no overlap; therefore, a between-group comparison of mean vernier acuity was conducted. The mean difference was calculated as the difference between the mean vernier acuity under baseline and SWIB conditions for each method. The mean difference varied significantly across all three methods (M1: 6.57 ± 9.03, M2: 274.31 ± 57.25, M3: 49.96 ± 15.46). The standard deviation was largest for M2, followed by M3, indicating high variability in the observed values around the mean.

### SWIB Quantification

**Figure 7:**
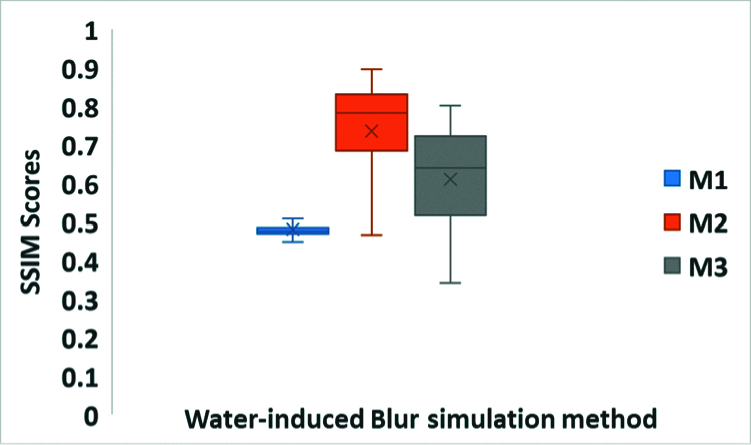
Box-Whisker-Plot of SSIM scores with all three water-induced blur simulation methods. The horizontal lines show the median, whereas the boxes indicate the interquartile range (IQR). Also, the “x” indicates mean SSIM scores, with whiskers denoting the maximum and minimum SSIM scores among three different water-induced blur simulation methods. M1, a glass tank filled with water; M2, a sprinkler with uncontrolled water splash; M3, a sprinkler with controlled water splash; SSIM, Structural Similarity Index Metric

The results suggest that with an increase in the magnitude of SB, SSIM also increased compared to images with SWIB. Figure 7 presents the mean, maximum, and range of SSIM scores observed with all three WIB simulation methods while comparing images with SB and SWIB. The results indicate that the maximum SB simulated using M1, M2, and M3 in our study were 1.5 D (SSIM: 0.71), 10 D (SSIM: 0.89), and M3: 4.5 D (0.80), respectively.

**Table 1:** Mean vernier acuity (arcsec) at baseline and SWIB conditions across three different water-induced blur simulation methods

**WIB simulation method**	**Baseline vernier acuity (mean ± std)**	**Vernier acuity under SWIB conditions (mean ± std)**	***P*** **-value (between baseline & SWIB)**	**Mean difference**
M1	13.28 ± 5.84*	19.85 ± 8.19 ∧	0.004	6.57
M2	14.44 ± 6.34*	288.75 ± 55.18 ∧	< 0.001	274.31
M3	14.05 ± 3.28*	64.01 ± 14.05 ∧	< 0.001	49.96
M1: A glass tank filled with water, M2: A sprinkler with uncontrolled water splash, M3: A sprinkler with controlled water splash; *P-value: 0.823 (one-way ANOVA for baseline vernier acuity among three different methods), ∧ *P*-value: < 0.001 (one-way ANOVA for vernier acuity under SWIB conditions across three different methods WIB, water-induced blur; SWIB, simulated water-induced blur).				

##  DISCUSSION

Stereo and vernier acuities, the two forms of hyperacuity, are in many aspects closely connected. Several studies have established a link between stereo acuity and vernier acuity, suggesting that they could be used to measure depth perception.^[31,32,33]^ Vernier acuity determines the relative position of visual features with a precision that exceeds the sampling resolution of cone receptors. It is thought to be mediated by orientation-tuned mechanisms and processed in the striate visual cortex. Studies have shown that vernier acuity improves with training.^[27]^ A study by Krauskopf and Farrell shows that, among other factors, luminance and blur are essential factors that adversely affect vernier acuity.^[34]^

The baseline vernier acuity obtained in the present study was comparable to that reported by Whitaker et al in normal healthy participants.^[35]^ As our results show, a significant decrease in vernier acuity under SWIB conditions represents a genuine change in sensitivity, rather than merely an effect of the learning curve. Randomizing the measurement of vernier acuity between baseline and SWIB conditions precludes any bias that could arise from learning. These results are further supported by Komori et al, who showed that depth perception is affected during swimming.^[36]^

The mean difference with M1 was significantly lower than with M2 and M3, which accounts for the absence of distortions with this WIB simulation method. Therefore, it is clear that the higher distortions caused by water will lead to more pronounced blur, which in turn affects visual functions. This shows that, similar to real-world environments, WIB can vary both spatially and temporally, and is not constant in any setup. Therefore, it is necessary to develop a blur metric specific to WIB in order to objectively qualify its effect on different visual functions while taking into account the amplitude, frequency, and oscillation properties of water waves.

A stimulus viewed through WIB varies because of the complex and diverse distortions caused by the water wave.^[37]^ Our results indicate a significant deterioration in vernier acuity across three WIB simulation methods. It is also noteworthy that the mean difference between the baseline and SWIB conditions was found to be higher in M2, followed by M3 and M1. This suggests that M2, compared to the other two methods, resulted in a more drastic reduction in vernier acuity in the presence of SWIB. In fact, since the SWIB was not controlled with M2, the distortions produced changes in both spatial (amplitude and frequency) and temporal (oscillation speed) aspects, resulting in a higher standard deviation. Compared to M2, M3 involved less distortion; therefore, the mean difference in vernier acuity between baseline and SWIB conditions was lower than that associated with M2.

The SWIB quantification analysis showed that the blur produced in this study was approximately 1.5 D, 10 D, and 4.5 D with the WIB simulation methods M1, M2, and M3, respectively. The vernier acuity was most deteriorated with M2 ( ∼ 274.31 arcsec), which produced the highest amount of blur (10 D). The second highest deterioration was associated with M3 ( ∼ 35.46.57 arcsec), which produced a blur of about 4.5 D. Lastly, M1 ( ∼ 6.57 arcsec) produced a blur of 1.5 D compared to baseline.

According to a previous study, decreased illumination and contrast may affect vision in the presence of WIB.^[38]^ Similarly, our results suggest that in the presence of WIB in any environment, one may experience difficulties with depth perception due to reduced illumination.

Human vision is a complex function, which is further complicated by the unpredictable underwater environment. We found that depth perception is altered in a controlled setting. This observation could have significant implications for swimming/diving performance and object localization underwater. Object localization is essential for underwater archaeologists/photographers/welders, marine engineers/biologists, and those active in underwater sports such as spearfishing and underwater hockey/football. Our results have significant implications for understanding individual performance in the areas where these individuals engage.

Our results suggest the impact of WIB on vernier acuity is not comparable across different WIB simulation methods owing to the varied spatial and temporal properties of these methods. The strength of the blur produced is also different across these methods. This finding highlights the need to develop a WIB-specific blur metric to objectively quantify the effect of blur on various visual functions. Similar to “diopter,” which is used to quantify optical blur, there should be a metric to quantify WIB that considers different properties, such as amplitude, frequency, and the strength of the blur, among others.

It will be interesting to study further the impact of turbidity and illumination on visual performance and how this performance adapts to WIB, as studies have shown that neural adaptation to visual blur can improve contrast sensitivity and visual acuity. Underwater training has helped children develop good underwater acuity, thanks to the combined effects of pupil constriction and strong accommodation. Future investigations should compare vernier acuity among swimmers, divers, and non-swimmers/divers to determine whether training or adaptation improves vernier acuity among individuals.

In summary, this study measured changes in vernier acuity across three different WIB simulation methods, revealing a significant decrease in vernier acuity due to the blur. This implies that swimmers and divers may experience difficulties with depth perception in an aquatic environment. However, the results were significantly different owing to the varied strength of blur across the three WIB simulation methods. Thus, a metric should be developed to objectively quantify WIB.

##  Financial Support and Sponsorship

This work was supported by the Science and Engineering Research Board's Start-up Research Grant (SRG/2019/000566) by the Government of India.

##  Conflicts of Interest

None.
